# Subjective experience of difficulty depends on multiple cues

**DOI:** 10.1038/srep44222

**Published:** 2017-03-13

**Authors:** Kobe Desender, Filip Van Opstal, Eva Van den Bussche

**Affiliations:** 1 Department of Psychology, Vrije Universiteit Brussel, Brussels, Belgium; 2 Center for Research on Cognition & Neurosciences (CRCN), Université Libre de Bruxelles (ULB), Belgium; 3 Department of Psychology, University of Amsterdam, The Netherlands

## Abstract

Human cognition is characterized by subjective experiences that go along with our actions, but the nature and stability of these experiences remain largely unclear. In the current report, the subjective experience of difficulty is studied and it is proposed that this experience is constructed by integrating information from multiple cues. Such an account can explain the tight relationship between primary task performance and subjective difficulty, while allowing for dissociations between both to occur. Confirming this hypothesis, response conflict, reaction time and response repetition were identified as variables that contribute to the experience of difficulty. Trials that were congruent, fast or required the same response as the previous trial were more frequently rated as easy than trials that were incongruent, slow or required a different response as the previous trial. Furthermore, in line with theoretical accounts that relate metacognition to learning, a three day training procedure showed that the influence of these variables on subjective difficulty judgments can be changed. Results of the current study are discussed in relation to work on meta-memory and to recent theoretical advancements in the understanding of subjective confidence.

A defining characteristic of human cognition is that our actions are accompanied by subjective experiences. For example, when pronouncing difficult words such as *Worcestershire sauce*, there is more to it than just the cognitive processes allowing its pronunciation. You will also have the experience that this particular word was difficult to pronounce and you will experience a sense of confidence in the correctness of your pronunciation. These subjective experiences are termed *metacognitive* since they are a reflection on other cognitive processes taking place.

Many different fields of research have adopted the term “metacognition” when studying how humans reflect on their behavior. For example, when deciding on the nature of a noisy perceptual input, we typically experience a sense of confidence in the accuracy of our decision^1^,^2^, or we become aware of errors in the decision process^3^. Likewise, when learning novel information, we sense whether we have effectively learned the newly acquired information^4^, and whether we will know the answer during recall^5^. Recently, the subjective experience of *response conflict* has attracted considerable attention^6^,^7^,^8^,^9^,^10^. Whenever deciding between two options, we have a subjective experience of difficulty associated with the resulting response. Some responses feel very easy to carry out, whereas others are experienced as more difficult. In experimental tasks, these experiences of difficulty are often studied by inducing conflict between potential actions. For example, in a priming task, participants might be asked to rapidly categorize a target arrow as pointing to the left or to the right. Shortly preceding this target, a prime arrow is flashed that either triggers the same response as the target (i.e., a congruent trial) or a different response, hence inducing conflict between the responses (i.e., an incongruent trial). Incongruent trials are more frequently rated as difficult compared to congruent trials. Thus, the conflict between potential responses is experienced as subjectively difficult^7^. This finding has been observed even with masked primes that were entirely invisible^8^,^9^,^11^, indicating that these difficulty ratings reflect genuine metacognitive experiences, rather than visual awareness of the conflict between prime and target.

Although the influence of response conflict on subjective difficulty has been documented, the *nature* and *stability* of the experience of difficulty remain unclear. Intuitively, one might expect that response conflict results in increased reaction times (RTs) and error rates, and that the subjective experience of difficulty results from this performance decrement. However, there is some recent evidence speaking against this possibility. In a previous study, the influence of response conflict on the experience of difficulty was still present in a subset of data in which congruent and incongruent trials were matched in terms of RTs^11^. This raises the intriguing possibility that a subjective experience of difficulty is *directly* based on the presence of response conflict. Based on repeated past experiences, participants might have learned that response conflict leads to actual errors in responding. Given that errors are experienced as aversive^12^, after repeated pairing, the presence of response conflict in itself might be experienced as aversive^13^. Thus, over time, participants might learn that the mere presence of response conflict is a good indicator of performance. After having learned this relation, response conflict could then be used as a *cue* for the construction of the subjective experience of difficulty, independent of task performance.

Importantly, from the assumption that response conflict can be learned to be an indicator for difficulty, it follows that any variable that is a good indicator for primary task performance, could act as a cue for the construction of subjective difficulty. Apart from response conflict, another variable that is clearly indicative of performance is reaction time (RT). Participants might have learned that this is a good proxy for task difficulty^14^. Finally, a third factor that will be considered is the possibility that subjective difficulty depends on expectations. In behavioral tasks, humans are inherently biased to expect responses to repeat over consecutive trials^15^. In serial two-choice RT tasks, for example, it has been observed that responses for stimulus repetitions are faster than responses for stimulus alternations^16^. Given that this variable is indicative of performance, trials with response repetitions might be experienced as subjectively easier than trials with response alternations. In sum, if subjective difficulty is based on cues that are indicative of performance, it should be possible to provide empirical evidence that these three variables affect judgments of difficulty.

While the previous concerns the construction of subjective difficulty, its *stability* is equally unclear. If subjective difficulty results from sampling information from multiple cues, to what extent is the relative contribution of these cues fixed? Theoretical work in the broader field of metacognition has already stressed the role of learning in the construction of metacognition^17^,^18^,^19^. For example, Pasquali and colleagues^17^ presented simulations of a neural network that learned to wager on its own responses. By learning which representations are indicative of good performance, the network was successful in evaluating its own accuracy. In this framework, metacognition arises because the brain continuously learns about its own activity^20^. Converging empirical work has documented that subjective certainty in a decision can be altered by means of training^21^,^22^,^23^. After training, participants are more confident in their decisions^23^, and better in discriminating their own errors from correct responses^22^. This theoretical emphasis on learning is in line with the current hypothesis that the brain first has to *learn* which variables are good indicators of performance, before they become cues that are used to construct subjective difficulty. Therefore, a second prediction of the current study is that the construction of subjective difficulty can be influenced by training participants to rely more on certain cues at the expense of others. By training participants that a certain cue is a highly reliable indicator of performance, it should gain more weight in the construction of subjective difficulty.

In a first experiment we will test if subjective difficulty judgments are constructed by integrating information from multiple cues. This will be done by examining if the three variables described above, namely response conflict, RT and response repetition, affect subjective difficulty judgments. A second experiment will investigate if the relative contribution of cues to subjective difficulty can be changed by training. Participants were trained to rely more on response conflict (Experiment 2a) or on RTs (Experiment 2b) as the main cue informing their subjective difficulty judgments.

## Experiment 1

### Participants

Thirty-one participants, 14 men, participated for monetary compensation (£ 15). Mean age was 24.3 years (*SD* = 5.2, range 19–42). All participants were right-handed and reported normal or corrected-to-normal vision. All experimental protocols were approved by the local ethics committee of the Vrije Universiteit Brussel. All methods of all experiments were performed in accordance with the relevant guidelines and regulations. In accordance with the approved guidelines, written informed consent was obtained from each participant prior to the experimental session. Non-overlapping results from this dataset have been published elsewhere^11^.

### Stimuli and apparatus

**Figure 1:**
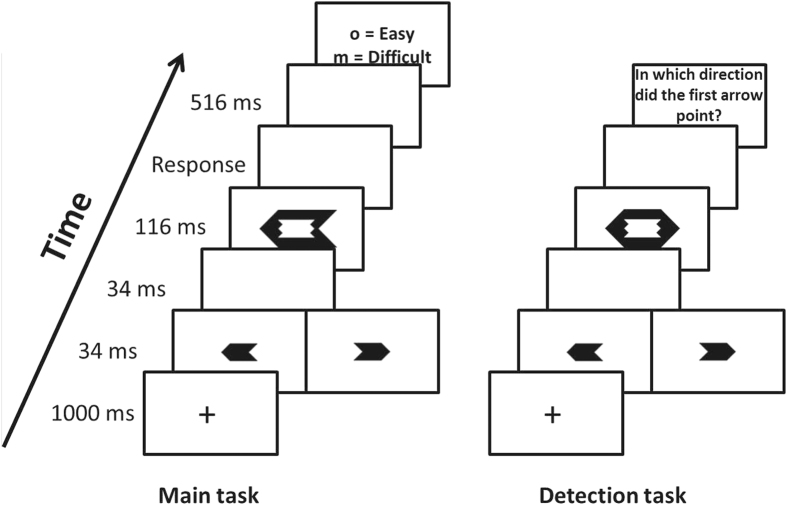
Example of a trial sequence of Experiment 1 in the main task (left) and in the detection task (right).

All stimuli were presented in white on a black background on a 15 inch CRT monitor, synchronized with a vertical refresh rate of 60 Hz. Experimental stimuli were prime arrows (1.5° wide and 0.7° high) and target arrows (3.3° wide and 1.4° high), that could point to the left or right (see Fig. 1). Because the prime arrows fitted perfectly within the contours of the target arrow (i.e., metacontrast masking^24^), primes were rendered invisible. Responses were collected using a standard QWERTY keyboard.

### Experimental procedure

Participants completed a masked priming experiment in which they additionally reported their metacognitive experience associated with each response. Each experimental trial started with a fixation cross for 1000 ms that was followed by a prime arrow for 34 ms, a blank screen for 34 ms, a target arrow for 116 ms, and finally a blank screen again. Participants were asked to respond as fast and accurately as possible to the direction of the target, by pressing “d” in response to a left pointing target arrow and “k” in response to a right pointing target arrow. They responded with the middle finger of each hand. If a response to the target was registered within 3000 ms, a blank screen was presented for 516 ms, followed by a screen asking participants about their subjective experience of difficulty: “How much difficulty did you experience when responding to the arrow? ”. They could answer either by pressing the “o” key with the ring finger of their right hand (“Rather more difficulty”) or by pressing the “m” key with the index finger of their right hand (“Rather less difficulty”). There was no time limit to answer this question. The inter-trial interval was 800 ms.

Each participant started with 20 practice trials in which the metacognitive question was omitted. Subsequently, the experimenter explained that participants had to rate their experience associated with a trial after each response. The experimenter motivated participants to use all information available to them (e.g., difficulty, error-tendency, response fluency) to answer this question. Participants were informed that there would be an equal amount of “more difficult” and “less difficult” trials, and they were motivated to keep a balance between these responses. Participants then received 20 additional practice trials with the metacognitive question. After these two training phases, each participant performed eight blocks of 80 trials each. In each block, half of the trials were congruent (i.e., prime and target pointing in the same direction), and half were incongruent (i.e., prime and target pointing in opposite directions).

To ensure that primes were genuinely invisible, participants performed a detection task after the main experiment. In this task, they were instructed to categorize the direction of the prime arrows, instead of the target arrows. During the detection task, targets were neutral with heads pointing in both directions to prevent that participants would respond to the target. It has been shown that these neutral targets provide a more sensitive test of prime visibility, compared to targets that are congruent or incongruent with the primes^25^. After the targets, a blank screen was presented for 516 ms, followed by a question about the prime direction. The detection task comprised 100 trials.

### Data analysis

The main aim of Experiment 1 was to examine how the variables response conflict, RT and response repetition affect primary task performance and subjective difficulty. Because RTs cannot be used as predictor in an analysis where they already serve as the dependent variable, they were omitted in the analysis of task performance. Analyses were done by fitting mixed effect models to our data. The most important advantage of this method is that it allows analyzing the effect of RTs on a trial-level. In traditional repeated measures approaches, one is obliged to create averages based on an arbitrary partitioning of the data (e.g., quartiles or tertiles), thereby losing an enormous wealth of information. Furthermore, mixed models are generally more powerful than traditional approaches to data analysis, better in handling unbalanced data which is insurmountable given the subjective nature of our task, and in analyzing categorical outcome variables^26^. Furthermore, error variance caused by between-subject differences can be accounted for by adding random slopes to the model. Although a full random effects structure has been suggested to be optimal^27^, this often results in overparameterized models that fail to converge^28^. Therefore, a model building strategy was used here. Random slopes were added for a variable only when this increased the model fit, as assessed by model comparison. To analyze RTs in responding to the target arrow, a linear mixed model approach was used, for which *F* statistics are reported and the degrees of freedom were estimated by Satterthwaite’s approximation, as implemented in the R library lmerTest^29^. To analyze metacognitive responses, a logistic linear mixed model approach was used. Significance of each variable and the interactions between variables in explaining the metacognitive response was assessed by computing *X^2^* statistics.

Model fitting was done in R^30^, using the lme4 package^31^. Interpretation of interaction effects was done by applying contrasts using the multcomp package^32^, or by computing fitted values using the effects package^33^.

## Results

### Primary task performance

To analyze reaction times, a linear mixed effects model was fitted predicting RTs on correct trials (96.7%), using equation (1), where X represents the fixed effects structure that is defined in equation (2), and Z the random effects structure defined in equation (3), for which the *b* is calculated for each participant i. The variables congruency (congruent or incongruent) and response repetition (repetition or alternation) were both factors with two levels.


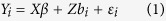



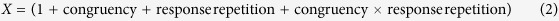



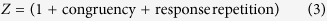


To deal with outliers, trials in which the RT deviated more than 3 *SD*s from their condition-specific mean were excluded from further analysis (i.e., 2.0%). Results showed that RTs were significantly faster on congruent trials (*M* = 457 ms) than on incongruent trials (*M* = 525 ms), *F*(1,30) = 123.70, *p* < 0.001. There was also an effect of response repetition, *F*(1,30) = 5.03, *p* = 0.032, reflecting faster RTs on response repetitions (*M* = 484 ms) compared to response alternations (*M* = 495 ms). There was no interaction between both, *F* < 1.

To analyze the error rates, a generalized mixed model as shown in equation (1) using a logistic link function was fitted to all data predicting accuracy (wrong or correct), using the same fixed and random effects structure as specified in equations (2) and (3), respectively. More errors were made on incongruent trials (*M* = 3.2%) than on congruent trials (*M* = 0.8%), *χ2*(1) = 35.37, *p *< 0.001. There was no main effect of response repetition, *p* = 0.45, nor an interaction between both, *p* = 0.58.

### Subjective difficulty

**Figure 2:**
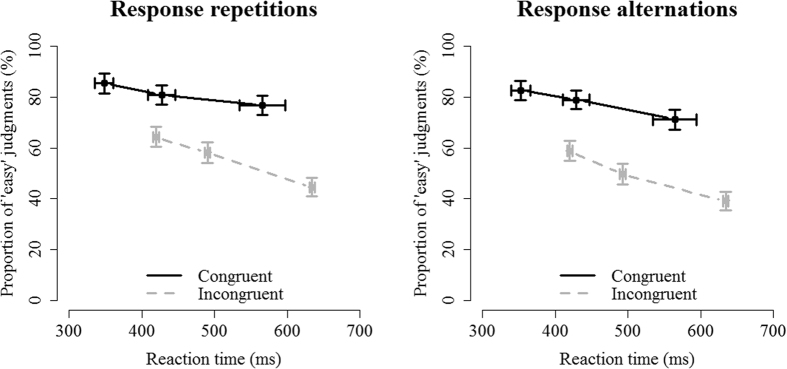
Results from Experiment 1. The proportion of ‘easy’ judgments are plotted as a function of RT (divided in three bins), congruency and response repetition. Participants generally judge congruent trials (black lines) as easier than incongruent trials (grey lines). Also, the proportion of ‘easy’ responses increases with decreasing RTs, which is visible in the negative slopes of the lines. Finally, response repetitions are overall judged to be easier than response alternations. Although not as noticeable as the other two effects, data in the left panel have slightly larger values on the y-axis compared to data in the right panel. Error bars reflect standard error of the mean.

To examine the influence of the three variables on subjective difficulty judgments, a generalized mixed model as shown in equation (1) using a logistic link function was fitted on correct trials predicting subjective difficulty judgments (easy or difficulty), using the fixed and random effects structures specified in equations (4) and (5), respectively. When the continuous predictor RT was entered as raw values, the fitted model was nearly unidentifiable, so to deal with this RTs were mean-centered and then scaled by the standard deviation, separately for each participant. To visually represent the findings, the proportion of ‘easy’ judgments is plotted as a function of the three independent variables in Fig. 2. To enhance visual interpretability, the data were divided in three bins, separately for each subject and each level of congruency.


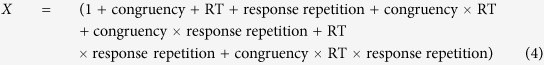



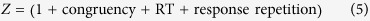


The analysis showed a main effect of congruency, *χ2*(1) = 19.33, *p *< 0.001, with a higher proportion of ‘easy’ judgments on congruent (*M* = 85.3%) compared to incongruent trials (*M* = 56.6%). In Fig. 2, this main effect can be derived from the fact that the black lines, reflecting congruent trials, lie above the grey lines, reflecting incongruent trials. There was also a main effect of RT, *χ2*(1) = 45.74, *p* < 0.001, with decreasing RTs leading to a decrease in the proportion of ‘easy’ judgments. This effect is reflected in the negative slopes in Fig. 2. Finally, there was a main effect of response repetition, *χ2*(1) = 16.61, *p* < 0.001, with a higher proportion of ‘easy’ judgments for response repetitions (*M* = 77.0%) compared to response alternations (*M* = 70.1%). Although not numerically as strong as the previous two effects, response repetitions have overall higher values on the y-axis (Fig. 2, left panel) compared to response alternations (Fig. 2, right panel). There was also a significant interaction between congruency and RT, *χ2*(1) = 6.39, *p* = 0.011, indicating that the effect of congruency on subjective difficulty is slightly larger when RTs are slower. No other effects were significant (all *p*’s > 0.13).

Because in Experiment 1, the instructions encouraged participants to use all information that was available to them, it is important to demonstrate that the current findings are not contingent on the exact instructions provided. Therefore, in the Supplementary Materials, we report the reanalysis of four additional studies that varied in small ways from Experiment 1, but that nevertheless produced highly similar results. In order to evaluate the effect of the instructions, the results of a replication study (reported first in the Supplementary Materials) are of most interest here. In that study participants only received the instructions that they had to decide on each trial whether their response felt rather easy or rather difficult, without any reference to which cue they had to use. Notwithstanding this difference in instructions and some other marginal differences, the results replicated those of Experiment 1: both congruency, reaction time and response repetitions reliably affected subjective difficulty judgments (see Supplementary Materials for full details).

### Prime visibility

To ensure that the effect of congruency on subjective difficulty does not simply reflect the visual resemblance between prime and target, we used the data of the detection task to compute *d*’ as an index of prime visibility. Left-pointing primes were treated as signal, right-pointing primes as noise. A left response to a left-pointing prime was considered a hit; the same response to a right-pointing prime was considered a false alarm. Hit and false alarm proportions were computed by dividing the total number of hits and false alarm by the number of signals. Results showed that *d*’ did not differ from chance level performance (i.e., zero), *d*’ = 0.10, *t*(30) = 1.12, *p* = 0.24, suggesting that primes were truly invisible.

## Experiments 2a and 2b

Experiment 1 showed that subjective difficulty judgments depend on multiple cues. First, trials were more frequently judged to be easy when they were congruent (compared to incongruent). Second, the proportion of ‘easy’ judgments linearly increased with decreasing RTs. Third, trials were more frequently judged to be easy when the response was a repetition of the previous trial (compared to an alternation). In Experiments 2a and 2b, it was tested whether the influence of these cues on the experience of difficulty can be changed by means of training. Participants were trained to rely more on response conflict (Experiment 2a) or RT (Experiment 2b) when providing their subjective difficulty judgment. Because response repetition is a very explicit cue once attention is directed towards it, it was not included in this metacognitive training. For this reason, and because the model did not converge when response repetition was entered as an additional factor into equation (8), this variable was excluded from further analysis.

### Participants

Twenty-three participants took part in Experiment 2a. Two were excluded because they made 52% and 55% errors in responding to the target arrow during the training. The final sample thus comprised 21 participants, three men, with a mean age of 20.2 years (*SD* = 2.2, range 18–25). All but one participant were right-handed. In Experiment 2b, 25 participants, seven male, took part. Mean age of the sample was 18.6 years (*SD* = 1.6, range 17–25). All but three participants were right-handed and participants reported normal or corrected-to-normal vision. Participants took part in return for course credits, and informed consent was obtained for all participants,

### Stimuli and apparatus

Stimuli and apparatus were identical to Experiment 1 except for the following. Because prime visibility might increase with practice of the task, the refresh rate was changed to 75 Hz to allow shorter prime durations.

### Experimental procedure

**Figure 3:**
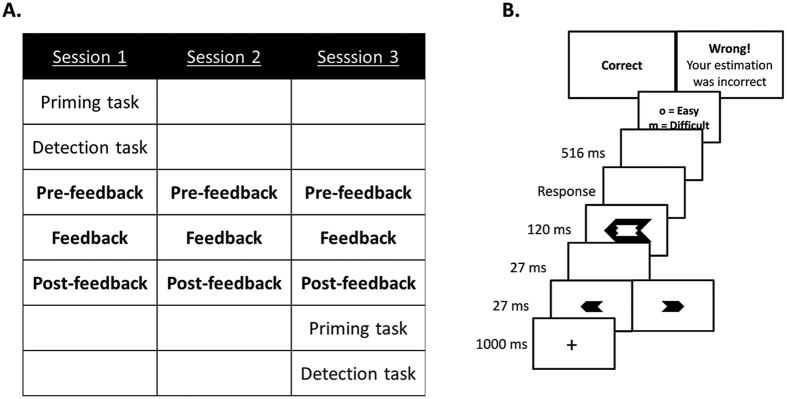
(**A**) Schematic overview of Experiments 2a and 2b, in which participants were trained to rely more on congruency and RTs, respectively, when providing their subjective difficulty judgment. In each training session (indicated in bold), participants performed three blocks of 98 trials that were identical to the design of Experiment 1. Except during the second block of each training session, participants received feedback on the accuracy of their subjective judgment. In the experimental analyses, the three different blocks were collapsed per session in order to examine how subjective difficulty ratings evolve over the three sessions. (**B**) Example of a trial in a feedback block.

Participants were invited to take part in three training sessions, which took place on three consecutive days. Figure 3 presents a schematic overview of the design of experiments 2a and 2b. In *session 1*, participants started with extensive training of the priming task without providing a subjective difficulty judgment. Each participant started with ten practice trials, followed by five blocks of 60 trials. Afterwards, participants performed a detection task in which they responded to the direction of the prime. The detection task comprised 150 trials: 50 trials with a neutral target as in Experiment 1, 50 trials in which the target was congruent with the prime and 50 trials in which it was incongruent. The neutral targets were used to obtain a maximally sensitive measure of prime visibility. For the non-neutral targets, it was predicted that participants who were trained to focus on congruency would be able to infer the prime direction based on their subjective experience of difficulty after training.

After the detection task, participants started with the subjective difficulty training. In Experiment 2a, participants were informed that due to the congruency between the invisible prime and the visible target, the task sometimes felt easy and sometimes difficult. They were told that they were going to be trained to become better in evaluating the difficulty of a trial, based on the prime-target congruency. It was explained that they could subjectively experience this conflict between both responses, and were instructed to primarily focus on this experience when providing their subjective difficulty judgment. In Experiment 2b, it was explained that sometimes RTs are rather slow which is typically experienced as difficult, and sometimes RTs are quite fast which is typically experienced as easy. They were motivated to use their RT as the main cue to base their subjective difficulty judgment on. Translated versions of the exact instructions can be found in the Supplementary Materials.

The subjective difficulty training started with eight practice trials. Then, participants performed 3 blocks of 98 trials (with a self-paced break each 48 trials), in which they first gave a speeded response to the target, followed by a judgment about their experience of difficulty on that trial. During the second block of each session, they received feedback during the inter-trial interval on the correspondence between their subjective judgment and the cue they were trained on. Specifically, in Experiment 2a, if participants judged a trial to be rather easy (rather difficult) on congruent (incongruent) trials, the feedback message read “Correct”. Otherwise, the feedback message read: “Wrong! Your estimation was incorrect”. In Experiment 2b, participants received feedback based on their RT. When the RT on a trial was faster (slower) than the mean RT of correct trials in the preceding block and participants judged this trial to be rather easy (rather difficult) the feedback message read “Correct”. Otherwise, the feedback message read: “Wrong! Your estimation was incorrect”. If participants responded incorrectly to the target arrow, it was said that no feedback could be provided because they made a mistake in their speeded response.

In *session 2 and session 3*, participants performed the same subjective difficulty training as in the first session. *Session 3* ended with the priming task followed by the detection task.

Experimental trials were identical to Experiment 1, except for the duration of the prime and blank, which was shortened to 27 ms, the duration of the target which now lasted 120 ms, and the duration of the ITI, which was prolonged to 1000 ms.

## Results

### The influence of training on primary task performance

To analyze RTs to the target arrow, correct trials (90.4%) were analyzed by fitting a linear mixed regression model predicting RTs using equation (1), with the fixed and random effects structure specified in equations (6) and (7), respectively. Session was a factor with three levels (first, second or third) and experiment a factor with two levels (2a or 2b). The data of the three blocks were collapsed per session. Trials with RTs more than 3 *SD*s from the condition-specific mean were excluded from the analysis (i.e., 2.0%).


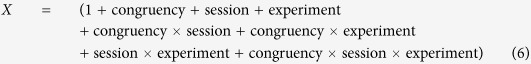



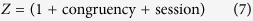


RTs were faster on congruent trials (*M* = 347 ms) compared to incongruent trials (*M* = 423 ms), *F*(1,44) = 519.38, *p* < 0.001. The congruency effect increased over sessions (session 1: *M* = 71 ms; session 2: *M* = 77 ms; session 3: *M* = 80 ms), *F*(2,34610) = 5.41, *p* = 0.003. Crucially, this increase did not differ between both experiments, *F* < 1, nor did the congruency effect differ between both experiments, *F*(1,44) = 2.99, *p* = 0.091. If anything, the congruency effect was numerically slightly larger in Experiment 2b (*M* = 81 ms) than Experiment 2a (*M* = 70 ms). RTs were overall faster in Experiment 2b (*M* = 343 ms) than in Experiment 2a (*M* = 426 ms), *F*(1,44) = 27.37, *p* < 0.001. RTs decreased over sessions, *F*(2,44) = 20.18, *p* < 0.001, and this effect was larger for Experiment 2a (session 1: *M* = 462, session 2: *M* = 430, session 3: *M* = 383) than Experiment 2b (session 1: *M* = 362, session 2: *M* = 335, session 3: *M* = 331), *F*(2,44) = 8.76, *p* < 0.001.

Error rates were examined by fitting a generalized mixed model as shown in equation (1) using a logistic link function on all data, predicting accuracy (wrong or correct), using the fixed effect structure specified in equation (6), and with a random structure that only contained random intercepts, but no random slopes. Error rates were 13.1% higher on incongruent trials (*M* = 14.7%) compared to congruent trials (*M* = 1.6%), *χ2*(1) = 1767.97, *p* < 0.001. Less errors were committed in Experiment 2a (*M* = 4.2%) than in Experiment 2b (*M* = 6.1%), *χ2*(1) = 12.47, *p* < 0.001, and error rates slightly increased over sessions (session 1: *M* = 4.3%, session 2: *M* = 5.5%, session 1: *M* = 5.7%), *χ2*(2) = 161.38, *p* < 0.001. This increase was different for Experiment 2a (session 1: *M* = 3.7%, session 2: *M* = 4.9%, session 1: *M* = 4.2%) than for Experiment 2b (session 1: *M* = 4.9%, session 2: *M* = 6.1%, session 3: *M* = 7.3%), *χ2*(2) = 8.87, *p* = 0.012. The congruency effect was smaller in Experiment 2a (*M* = 7.1%) than in Experiment 2b (*M* = 20.2%), *χ2*(1) = 154.51, *p* < 0.001. The congruency effect increased over sessions (session 1: *M* = 9.3%, session 2: *M* = 14.1%, session 3: *M* = 16.8%), χ2(2) = 23.46, *p* < 0.001. Finally, there was a three-way interaction between all three variables, χ2(2) = 14.10, *p* < 0.001. The increase in congruency effect was much less pronounced in Experiment 2a (session 1: *M* = 5.4%, session 2: *M* = 6.5%, session 3: *M* = 9.5%) compared to Experiment 2b (session 1: *M* = 13.6%, session 2: *M* = 22.9%, session 3: *M* = 25.5%).

### Training subjective difficulty

To examine whether it is possible to change the influence of the identified cues by means of training, a generalized mixed model as shown in equation (1) using a logistic link function was fitted on correct trials, predicting the subjective difficulty judgment (easy or difficulty) using the fixed effects structure specified in equation (8) and the random effects structure in equation (9).


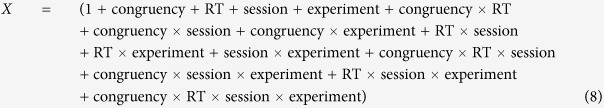



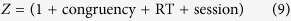


In line with Experiment 1, there was a main effect of congruency, *χ2*(1) = 17.64, *p *< 0.001, with a higher proportion of ‘easy’ judgments on congruent (*M* = 84.9%) compared to incongruent (*M* = 72.9%) trials. There was also a main effect of RT, *χ2*(1) = 180.92, *p* < 0.001, with faster RTs leading to an increase in the proportion of ‘easy’ judgments. There also was a main effect of experiment, *χ2*(1) = 31.83, *p* < 0.001, showing that participants were less biased towards ‘easy’ responses in Experiment 2a, *M* = 70.6%, compared to Experiment 2b, *M* = 86.4%. Contrary to Experiment 1, there was no interaction between congruency and RT, *p* = 0.52. Although there was a significant three-way interaction with session, *χ2*(2) = 8.30, *p* = 0.016, in none of the three sessions there was a significant interaction between congruency and RT (session 1, *z* = 1.17, *p* = 0.24; session 2, *z* = −0.23, *p* = 0.82; session 3, *z* = −0.73, *p* = 0.47). There were further significant interactions between congruency and session, *χ2*(2) = 12.92, *p* = 0.001, RT and session, *χ2*(2) = 8.65, *p* = 0.013, congruency and experiment, *χ2*(1) = 12.90, *p* < 0.001, RT and experiment, *χ2*(1) = 51.69, *p* < 0.001, and session and experiment, *χ2*(2) = 6.82, *p* = 0.033. The most important question here, however, is whether the main effect of congruency and the main effect of RT change over session, and whether this change was different for both experiments. Confirming this prediction, there were significant three-way interactions between congruency, session and experiment, *χ2*(2) = 37.84, *p* < 0.001, and between RT, session and experiment, *χ2*(2) = 76.47, *p* < 0.001. To interpret these higher-order interactions, the data were further analyzed for each experiment separately.

**Figure 4:**
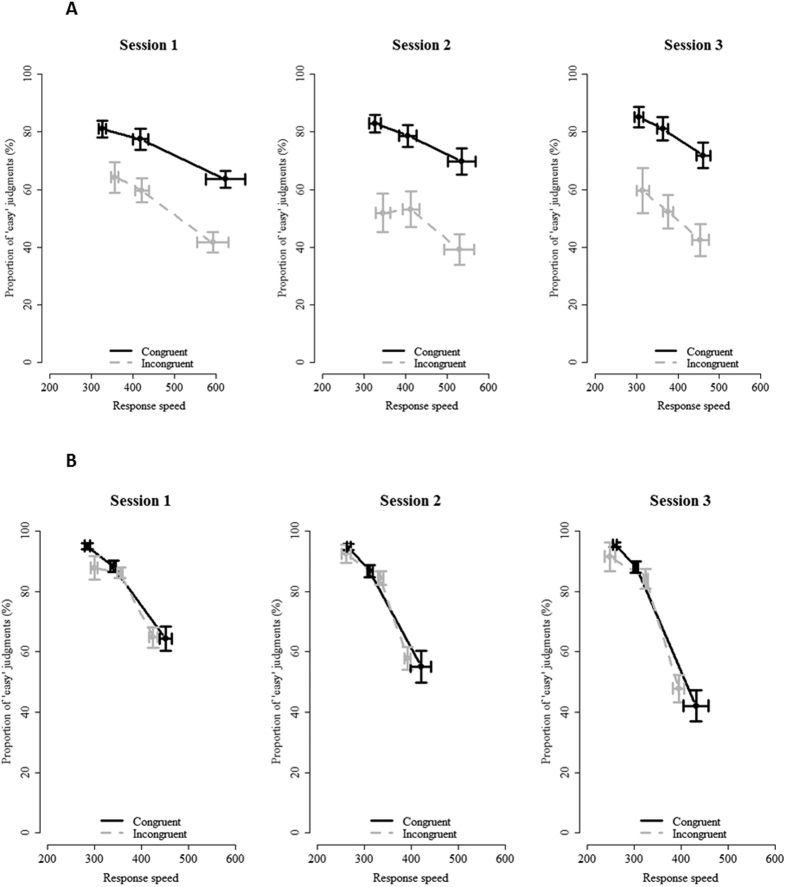
Results of subjective difficulty training. (**A**) In Experiment 2a, participants were trained to primarily use congruency as a cue. As indicated by an increasing distance between congruent (black) and incongruent (grey) lines, the influence of congruency on subjective difficulty judgments indeed increased over sessions. (**B**) In Experiment 2b, participants were trained to use RT as a cue. Over sessions, the effect of RT on subjective difficulty judgments indeed increased, as evidenced by the increasing steepness of the slopes. Error bars reflect standard error of the mean.

In Experiment 2a, the predicted interaction between congruency and session was found, *χ2*(2) = 45.43, *p* < 0.001, indicating that the influence of congruency on subjective difficulty judgments increased over sessions. This effect is reflected in Fig. 4A as an increasing distance between the congruent (black) and incongruent (grey) lines. Specifically, the difference in proportion of easy judgments on congruent versus incongruent trials increased over sessions: 21.7% on session 1 (congruent: 79.4%, incongruent: 57.7%), 32.7% on session 2 (congruent: 81.7%, incongruent: 49.0%), and 34.4% on session 3 (congruent: 84.7%, incongruent: 50.3%). The interaction between RT and session was also significant, *χ2*(2) = 13.93, *p* < 0.001. The effect of RT on subjective difficulty judgments remained constant from session 1 (*β* = −0.85, *z* = 7.19, *p* < 0.001) to session 2 (*β* = −0.87, *z* = 5.32, *p* < 0.001), and then decreased on session 3 (*β* = −0.71, *z* = 3.88, *p* < 0.001). The three-way interaction was not significant, *χ2*(2) = 5.02, *p* = 0.08.

In Experiment 2b, there was a significant interaction between RT and session, *χ2*(2) = 68.74, *p* < 0.001, showing that, as hypothesized, over sessions participants relied more on RT when providing their subjective difficulty judgments (session 1: *β* = −1.90, *z* = −9.83, *p* < 0.001, session 2: *β* = −2.30, *z =* −8.18, *p* < 0.001, session 3: *β* = −3.26, *z* = −9.43, *p* < 0.001). In Fig. 4B, it can be seen that the relation between RT and subjective difficulty judgments becomes stronger over sessions, as evidenced by an increasing steepness of the slope over sessions. There also was an interaction between congruency and session, *χ2*(2) = 6.90, *p* = 0.032, showing that the influence of congruency on subjective difficulty judgments *decreased* over sessions. The difference in proportion of easy judgments between congruent and incongruent trials was 3.1% on session 1 (congruent: 89.6%, incongruent: 86.5%), 0.4% on session 2 (congruent: 87.5%, incongruent: 87.1%), and 0.4% on session 3 (congruent: 83.6%, incongruent: 83.2%). There was no three-way interaction, *p* = 0.21.

### The role of instructions versus feedback

Because the model depicted in equation (8) did not converge when block (first block, feedback block, third block) was entered as an additional factor, it was not possible to examine how the influence of congruency and RT on subjective difficulty judgments is shaped by the feedback that is provided during the feedback block. To still be able to examine this question, the analyses reported above were repeated, but now separately for each session, which allows entering block as a factor. More specifically, subjective difficulty judgments (easy or difficulty) on correct trials were fitted using the fixed effects structure specified in equation (10) and the random effects structure in equation (11), separately for the data of each session. Block was added as a factor with three levels: first block, feedback block and third block. An additional advantage of this analysis, is that it is possible to examine the first block of the first session (i.e., before any feedback was given), where it can be tested whether the effect of congruency and RT on subjective difficulty judgments already differ solely based on the instructions.


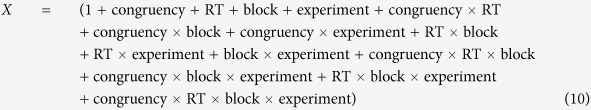



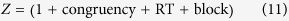


#### First session

Apart from previously reported main effects of congruency, RT, and experiment (all *p*’s < 0.001), the first session showed a main effect of block that just failed to reach significance, *χ2*(2) = 5.05, *p* = 0.08. The proportion of ‘easy’ judgments varied over blocks (block 1: *M* = 84.4%, feedback block: *M* = 80.2%, block 3 = 82.1%). The data further replicated previous analyses showing that the effect of congruency was larger in Experiment 2a (*M*_congruent_ = 78.7%, *M*_incongruent_ = 58.6%) than in Experiment 2b (*M*_congruent_ = 90.2%, *M*_incongruent_ = 88.1%), *χ2*(1) = 6.81, *p* = 0.009, and that the effect of RT was larger in Experiment 2b than in Experiment 2a, *χ2*(1) = 20.71, *p* < 0.001. Importantly, there was no interaction between congruency, block and experiment, *p* = 0.14. Follow-up analyses showed that the effect of congruency on subjective difficulty judgments did not differ between blocks, neither in Experiment 2a, *p* = 0.24, nor in Experiment 2b, *p* = 0.23. Interestingly, there was a significant interaction between reaction time, block and experiment, *χ2*(2) = 23.71, *p* < 0.001. In Experiment 2b, there was a significant interaction between RT and block, *χ2*(2) = 29.077, *p *< 0.001, showing that the effect of RT on subjective difficulty judgments increased from the first block (*β* = −1.41, *χ2*(1) = 38.61, *p *< 0.001) over the feedback block (*β* = −2.44, *χ2*(1) = 70.11, *p *< 0.001) to the third block (*β* = −2.59, *χ2*(1) = 78.88, *p *< 0.001). In Experiment 2a, there also was an interaction between RT and block, *χ2*(2) = 7.51, *p* = 0.023. The effect of RT on subjective difficulty judgments decreased from the first block (*β* = −0.97, *χ2*(1) = 30.25, *p *< 0.001) to the feedback block (*β* = −0.54, *χ2*(1) = 25.57, *p *< 0.001) and then increased again in the third block (*β* = −1.07, *χ2*(1) = 32.40, *p *< 0.001).

A final comparison showed that already in the first block of the first session (i.e., before any feedback was presented), the effect of congruency on subjective difficulty judgments differed between Experiment 2a (*M* = 19%) and Experiment 2b (*M* = 5.99%), *χ2*(1) = 4.72, *p* = 0.03, whereas the effect of RT on subjective difficulty judgments did not differ between both experiments, *p* = 0.19. This shows that the instructions provided to participants at the start of the experiment influenced the effect of congruency on subjective difficulty judgments, whereas they did not significantly influence the effect of RTs.

#### Second session

Apart from previously reported main effects of congruency, RT, and experiment (all *p*’s < 0.001), the second session showed a main effect of block, *χ2*(2) = 30.16, *p* < 0.001. The proportion of ‘easy’ judgments varied over blocks (block 1: *M* = 87.3%, feedback block: *M* = 75.2%, block 3 = 80.0%). The data further replicated previous analyses showing that the effect of congruency was larger in Experiment 2a (*M*_congruent_ = 83.8%, *M*_incongruent_ = 48.5%) than in Experiment 2b (*M*_congruent_ = 87.7%, *M*_incongruent_ = 88.7%), *χ2*(1) = 15.72, *p *< 0.001, and that the effect of RT was larger in Experiment 2b than in Experiment 2a, *χ2*(1) = 47.20, *p* < 0.001. Most crucially, there was no significant three-way interaction between block, RT and experiment, *p* = 0.13. Although there was a significant interaction between block, congruency and experiment, *χ2*(2) = 6.29, *p* = 0.043, follow-up analyses did not reveal a significant modulation of congruency by block, neither in Experiment 2a, *p* = 0.19, nor in Experiment 2b, *p* = 0.11.

#### Third session

Apart from previously reported main effects of congruency, RT, and experiment (all *p*’s < 0.001), the data of the third session showed a main effect of block, *χ2*(2) = 12.87, *p* < 0.001. The proportion of ‘easy’ judgments varied over blocks (block 1: *M* = 82.8%, feedback block: *M* = 75.1%, block 3 = 82.1%). The data further replicated previous analyses showing that the effect of congruency was larger in Experiment 2a (*M*_congruent_ = 87.4%, *M*_incongruent_ = 50.1%) than in Experiment 2b (*M*_congruent_ = 84.1%, *M*_incongruent_ = 84.6%), *χ2*(1) = 15.25, *p* < 0.001, and that the effect of RT was larger in Experiment 2b than in Experiment 2a, *χ2*(1) = 53.45, *p* < 0.001. Importantly, there was no significant interaction between congruency, block and experiment, *p* = 0.10, and only a marginally significant interaction between RT, block and experiment, *χ2*(2) = 6.00, *p* = 0.050. Follow-up analyses revealed that the effect of RT on subjective difficulty ratings increased over the three blocks in Experiment 2b, *χ2*(1) = 7.05 *p* = 0.029 (block 1: *β =* −2.34, *χ2*(1) = 67.98, *p* < 0.001; feedback block: *β* = −3.45, *χ2*(1) = 100.53, *p* < 0.001; block 3: *β* = −3.71, *χ2*(1) = 66.30, *p* < 0.001), whereas it did not in Experiment 2a, *p* = 0.56.

In sum, these additional analyses demonstrate that while the feedback seemed to be especially helpful to take RT into account when providing subjective difficulty judgments, the changing influence of congruency on the subjective difficulty judgment was mainly caused by the verbal instructions, as evidenced by the fact that the effect of congruency on subjective difficulty judgments already differed depending on the instructions in the first block of the first session, before any feedback was provided.

### Prime visibility

**Figure 5:**
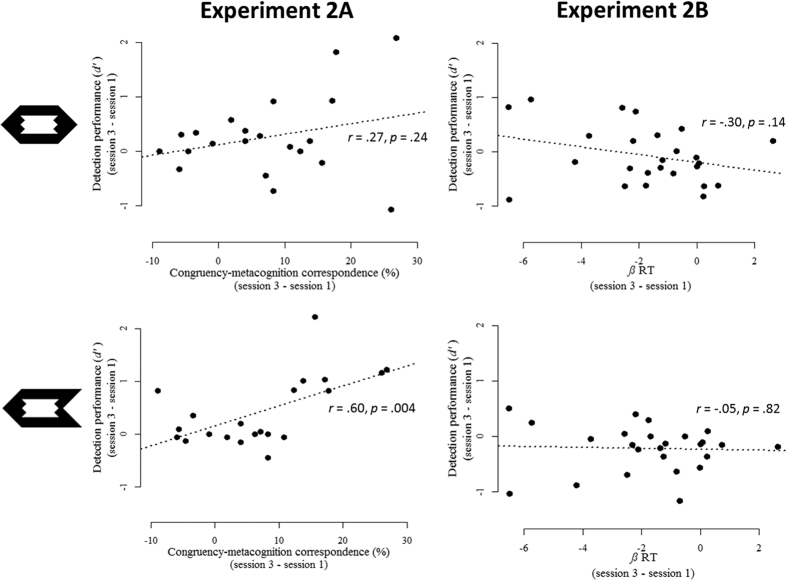
Detection performance in Experiments 2a and 2b. Top row: when neutral targets are used in a detection task, detection performance (*d*’) indicates the true level of prime visibility. In both experiments, increases in detection performance were unrelated to increased reliance on the trained cue. Bottom row: when left or right pointing targets are used during the detection task, it was hypothesized that participants can use their subjective difficulty experience to infer the direction of the prime. Indeed, this was the case for participants trained to focus on congruency (left) but not for participants trained on RT (right).

To exclude the possibility that the increasing influence of congruency simply reflects an increase in prime visibility, our measure of prime visibility, *d*’, was computed once based on the detection data of *session 1* and once on the detection data of *session 3*. Similar to Experiment 1, to obtain a true measure of prime visibility, *d*’ was calculated only on trials with neutral targets^25^. In Experiment 2a, prime visibility was slightly above chance level performance in the first session (*d*’ = 0.24), *t*(20) = 2.19, *p* = 0.04, and in the third session (*d*’ = 0.51), *t*(20) = 2.85, *p* = 0.01. The numerical increase between both was not significant, *t*(20) = −1.65, *p* = 0.12. Furthermore, there was no relation between the training effect on congruency (session 3–session 1) and this non-significant increase in prime visibility, *r* = 0.27, *p* = 0.24. In Experiment 2b, prime visibility was not different from chance level performance in the first session (*d*’ = 0.13), *t*(24) = 1.54, *p* = 0.13, nor in the third session (*d*’ = 0.06), *t*(24) = 0.59, *p* = 0.56. This numerical decrease in prime visibility was not significant, *t*(24) = 0.62, *p* = 0.54. There was no relation between the increased influence of RT on subjective difficulty judgments, computed by subtracting the *β* of session 3 from the *β* of session 1, and this decrease in prime visibility, *r* = −0.30, *t*(23) = −1.52, *p* = 0.14 (see Fig. 5).

Next, we also computed *d*’ based on the detection trials in which targets were pointing to the left or right. It was predicted that participants should become better in detecting the prime here, because they can infer the prime direction based on their subjective experience, however only when they are trained on congruency (i.e., in Experiment 2a). In Experiment 2a, in the first session the *d*’ value was 0.18, *t*(20) = 2.36, *p* = 0.028, and the third session it was 0.61, *t*(20) = 3.37, *p* = 0.003. This increase from session 1 tot session 3 was indeed significant, *t*(20) = −3.01, *p* = 0.007. As predicted, the larger the training effect on congruency (session 3–session 1), the larger the increase in *d*’, *r* = 0.60, *p* = 0.004. Thus, even when participants are not rapidly responding to the target, they seem to experience congruent trials as easy and incongruent trials as difficult, and they can use this experience to infer the direction of the subliminal prime. Note, however, that this latter correlation of *r* = 0.60 (Fig. 5, lower left) was not significantly larger than the correlation of *r* = 0.27 (Fig. 5, upper left) obtained with neutral target arrows, *t* = 1.36, *p* = 0.19. Therefore, it cannot be claimed that both correlations significantly differ depending on the target arrow. In Experiment 2b, there were also differences in *d*’ between the first session (*d*’ = 0.25), *t*(24) = 3.12, *p* = 0.004, and the third session (*d*’ = 0.04), *t*(24) = 0.49, *p* = 0.63. Unexpectedly, this *decrease* was significant, *t*(24) = 2.54, *p* = 0.018. This decrease in *d*’, however, was unrelated to the increasing influence of RT on subjective difficulty judgments, *r* = −0.04, *t*(23) = −0.22, *p* = 0.82.

## General Discussion

The current study showed that the subjective experience of difficulty depends on multiple cues that are indicative of performance. In Experiment 1, it was shown that response conflict, reaction time, and response repetition influenced subjective difficulty judgments. Trials that were congruent, fast or required the same response as the previous trial were more frequently rated as easy compared to trials that were incongruent, slow or had alternating responses. Moreover, it was shown that the relative contribution of these cues can be changed by means of training: training participants to rely more on congruency (Experiment 2a) or reaction time (Experiment 2b) for their subjective difficulty judgment increased the influence of this cue on their judgment. Given that subjective judgments of difficulty reflect the subjective appreciation of performance, such judgments are typically classified as *metacognitive*. In the remainder we will discuss commonalities and differences between the construction of subjective difficulty and related metacognitive constructs, such as judgments of learning and confidence ratings.

### Subjective difficulty versus judgment of learning (JOL)

The empirical findings of the current work nicely converge with theoretical work in meta-memory research. In a typical study on meta-memory, participants learn the association between two arbitrarily paired words. At the end of each learning trial, they provide a judgment of learning (i.e., JOL), which indicates their believed likelihood of remembering one word of a pair when presented with the other. By examining how well these JOLs track the actual memory performance, participants’ ability to monitor their own learning can be studied^34^,^35^. Interestingly, these JOLs are not constructed based on the actual strength of the memory trace, but rather reflect the integration of multiple cues. For example, Koriat^35^ argued that JOLs are determined by internal cues (e.g., difficulty of the material), external cues (e.g., study time), and mnemonic cues, such as familiarity of the answer^5^, and memory for past performance^4^. The extent to which JOLs are good predictors of actual performance thus depends on the empirical correlation between these cues and actual memory performance. It is clear that this account bares close resemblance to the mechanisms underlying subjective difficulty. For example, in Experiment 1 of the current work, the influence of response repetitions on subjective difficulty was highly significant, whereas its influence on task performance barely reached significance. This finding is easily explained by assuming that subjective difficulty is *directly* based on the occurrence of a response repetition (i.e., on the presence of this cue), not on the effect it has on performance.

Interestingly, Koriat also showed that the construction of JOLs changes with learning. With training, JOLs become more dependent on mnemonic factors and less on external factors. Given that the former are often better predictors of performance than the latter, this change in relative contribution of both cues can explain the increased correlation between JOLs and memory performance with practice^35^. An important difference with such a training procedure, however, is that in the current study participants were trained to rely more on a specific cue, instead of acquiring more experience with the task. This, however, also raises the important question as to whether our training procedure altered the actual subjective experience of difficulty, or whether participants simply complied with the instructions to rely more on the indicated cue, without actually experiencing this. Another important factor that should be taken into account, are the precise mechanisms that led to a change in the construction of subjective difficulty judgments. In comparing Experiments 2a and 2b, there was suggestive evidence that the increasing influence of different cues across sessions was caused by different mechanisms. The increasing influence of congruency on subjective difficulty judgments seemed to be largely driven by instructions. Already in the first block of the first session (i.e., before any feedback was presented), the influence of congruency on subjective difficulty judgments was larger in Experiment 2a than in Experiment 2b. This shows that the mere instruction to take congruency into account is sufficient to do so. Moreover, there was no indication that the feedback was effective in increasing the influence of congruency. The results of Experiment 2b were opposite: whereas instructions did not have a clear effect (i.e., the influence of RT did not differ between the first block of the first session of both experiments), the feedback was very effective. In Experiment 2b, the influence of RT on subjective difficulty judgments always increased during and after feedback was presented. A challenge for future work will be to further unravel why and how different mechanisms shape subjective difficulty judgments, and how these relate to the influence of learning on other metacognitive judgments, such as the training effect on JOLs reported above.

### Subjective difficulty versus subjective confidence

In contrast to the clear commonalities between our findings and those in the field of meta memory, they are much less consistent with recent developments in the understanding of subjective confidence^36^,^37^,^38^. For example, in a simple visual discrimination task, it was shown that confidence judgments carefully track performance, rather than reflecting the integration of the available cues^37^. Findings like these have led to a normative interpretation, according to which subjective confidence is constructed based on the same evidence that led to the decision^17^,^39^. Consider, for example, deciding whether a faint Gabor patch is tilted left or right. Evidence for each decision option can be thought of as the firing rate of orientation-specific neurons tuned to these directions. The pool of neurons whose activation first reaches a decision threshold determines the choice^40^. Subjective confidence in this decision can be computed by comparing evidence favoring the chosen option to evidence favoring the unchosen option^41^,^42^, or selectively by the strength of the evidence of the chosen option^43^. Critically, since confidence is based on (a transformation of) the evidence that also influenced the decision, these accounts predict a close correspondence between accuracy and confidence. At this point, work on subjective confidence clearly differs from both the current work and the meta-memory literature, where such dissociations naturally arise if cues are not predictive of actual performance. It should be noted, however, that there is also growing evidence that confidence and accuracy can be clearly dissociable, which is incompatible with a normative view. For example, dissociations between confidence and accuracy have been observed by manipulating attention^44^, by varying the degree of variability in the signal^45^, or by applying transcranial magnetic stimulation (TMS) to the prefrontal^46^ or premotor^47^ cortex. Thus, although normative models provide the dominant interpretation of how confidence judgments are constructed, there is growing evidence pointing towards possible dissociations between confidence and accuracy.

One major conclusion from the discussion above is that studies in the field of metacognition should be very precise about their object of study. Although metacognitive judgments share the feature that they are a reflection upon performance, there are large differences in the underlying computational processes that gave rise to different judgments. Further cross-fertilization between these fields might aid to shed more light on the differences and commonalities of the rich variety of metacognitive experiences. For example, in a standard perceptual decision making task, it was observed that the variability of perceptual input affects subjective confidence more than it influences primary task performance^45^. One potential explanation for this finding is that participants use the variability of information as a cue for their confidence judgments.

### Other cues of difficulty

The scarce research that has focused on subjective difficulty has mainly examined the role of response conflict^7^,^8^,^9^,^11^. One of the major contributions of the current work is the demonstration that subjective difficulty not only depends on response conflict, but on multiple cues. Although we have put forth response conflict, reaction time and response repetitions as cues, any variable that is indicative of primary task performance could potentially be used as a cue. For example, in Experiment 1, responses to the target were slightly faster with the left hand (*M* = 470 ms) than with the right hand (*M* = 495 ms), *F*(1,27) = 6.67, *p* = 0.015, which probably resulted from the fact that judgments of subjective difficulty were always given with the right hand. Given that response hand is thus indicative of performance, it comes as no surprise that left-hand responses were also more frequently judged to be easy (*M* = 76.1%) than right-hand responses (*M* = 71.0%), *χ2*(1) = 36.95, *p *< 0.001. Importantly, this effect did not influence the other three main effects reported above (all *p*’s < 0.001). Ruling out alternative explanations, we examined the role of response hand in the replication study (*N* = 27) that is reported in the Supplemental Materials. As already noted, in this study judgments of difficulty were given with the *left* hand. Interestingly, the results were identical to Experiment 1, except for this specific aspect: Responses were now slower with the left hand (*M* = 470 ms) than with the right hand (*M* = 440 ms), *F*(1,25.91) = 9.83, *p* = 0.004, and left-hand responses were *less* frequently judged to be easy (93.2%) than right-hand responses (96.2%), *χ2*(1) = 15.88, *p* < 0.001. As this example nicely illustrates, subtle variations in the experimental design of a study that consistently affect performance, also have a robust effect on subjective difficulty.

### Can subjective difficulty be used to infer the direction of a masked prime?

One of the additional efforts of the current work was to examine the possibility that participants can use their experience of subjective difficulty to infer the direction of a masked prime. As recommended in the literature^25^, a detection task was carried out using a neutral target to ensure that primes are truly invisible. Yet, participants also carried out a detection task in which arrow targets pointing left or right were used, to test another hypothesis. It has been shown that response conflict is detected by the brain, even when participants simply view the screen without responding^48^. Thus, it could be that participants also have an experience of subjective difficulty during the detection task based on the mismatch between prime and arrow target, even though they do not respond to the targets. Interestingly, if this is the case it could be that participants use this experience of subjective difficulty to infer the direction of the masked prime (e.g., ‘this felt like a difficult trial, so the direction of the prime was probably opposite to the arrow target’). Both for Experiment 2a and 2b, *d*’ (i.e., a measure of detection performance) obtained with neutral targets was not different before and after training. Because this reflects a true measure of prime visibility, it shows that the visibility of the prime itself did not increase with training. For the data obtained with non-neutral arrow targets, results were different. Participants trained to rely more on congruency (Experiment 2a) showed a larger *d*’ after training. Moreover, this increase in *d*’ showed a positive correlation with the training effect itself: the larger the increase in relying on congruency when providing a difficulty judgment, the larger the increase in *d*’. Both these findings were not observed in the group of participants trained to use RT as a cue (Experiment 2b). Because this cue is not available during the detection task, the absence of the effect in this group is expected. However, as already reported, the improvement in the condition with non-neutral targets (e.g., Fig. 5 lower left panel) was not significantly larger than that in the condition with neutral targets (e.g., Fig. 5 upper left panel). Given that this is crucial to infer differences between conditions^49^, future research should aim to shed light on the robustness of this finding.

## Conclusion

In the current study, evidence was provided that subjective difficulty is constructed based on multiple cues. It was further shown to be possible to train participants to increasingly rely on a specific cue when providing a subjective difficulty judgment.

## Additional Information

**How to cite this article**: Desender, K. *et al*. Subjective experience of difficulty depends on multiple cues. *Sci. Rep.* **7**, 44222; doi: 10.1038/srep44222 (2017).

**Publisher's note:** Springer Nature remains neutral with regard to jurisdictional claims in published maps and institutional affiliations.

## Supplementary Material

Supplementary Information
