# Supplementary material for: Subjective experience of difficulty depends on multiple cues
Source: Sci Rep. 2017 Mar 13;7:44222. doi: 10.1038/srep44222 (PMC5347002; doi:10.1038/srep44222)
Supplement: Supplementary Information [file srep44222-s1.pdf]

Subjective experience of difficulty depends on multiple cues

Kobe Desender, Filip Van Opstal, & Eva Van den Bussche

1. Reanalyses of previous studies

In Experiment 1, it was demonstrated that subjective difficulty ratings are influenced by the variables congruency, reaction time and response repetition. To demonstrate the general nature of these findings, independent from the specific instructions or design features, we report the reanalysis of four additional studies that varied in small ways from Experiment 1, but nevertheless produced highly similar results.

First, we report the results of a replication study (*N* = 27) which was identical to Experiment 1, except for the following: The experiment lasted only 100 trials, participants provided a judgment of difficulty by pressing either "1" or "2" with their *left* hand, and the refresh rate was switched to 85hz in order to allow slightly shorter presentations of the prime (11.6 ms) and the blank (23.5 ms). Most crucially, participants only received the instructions that they had to decide on each trial whether their response felt rather easy or rather difficult, without any reference to which cue they had to use. The data of one participant was excluded because mean accuracy was below 80%, and that of another participant because the median RT of correct trials was more than 3 SDs above the group mean. Notwithstanding these marginal differences, the results replicated those of Experiment 1: Congruent trials were more frequently judged to be easy (*M* = 95.8%) than incongruent trials (*M* = 92.9%), $\chi2(1) = 18.13$, $p < .001$. The proportion of 'easy' responses was higher on response repetitions (*M* = 95.8%), than response alternations (*M* = 93.1%), $\chi2(1) = 12.96$, $p < .001$. Finally, the proportion of 'easy' judgments increased with decreasing RTs, $\chi2(1) = 126.93$, $p < .001$. No other effects were significant, all *p*'s > .24.

Second, in a published dataset [1], the instructions to participants were to judge on each trial whether a trial was conflicting or not. This judgment was made on a four-point scale with the left hand. Because participants hardly used options 2 and 3, this analysis was restricted to responses 1 and 4 (see [1], for more details). As reported in the paper, this study used pattern masking rather than metacontrast masking to render the primes invisible. While in this study the instructions specifically encouraged participants to focus on response conflict, the same pattern of results was obtained: Congruent trials were more frequently judged to be easy (*M* = 77.3%) than incongruent trials (*M* = 36.4%), $\chi2(1) = 86.04$, $p < .001$. Also, the proportion of 'easy' responses was higher on response repetitions (*M* = 69.3%), compared to response alternations (*M* = 48.5%), $\chi2(1) = 64.28$, $p < .001$. Finally, the proportion of 'easy' judgments increased with increasing RTs, $\chi2(1) = 46.36$, $p < .001$. Similar to Experiment 1, the interaction between congruency and RT was significant, $\chi2(1) = 5.54$, $p = .018$. No other effects were significant, all *p*'s > .22.

In a third unpublished dataset (*N* = 30), participants were told to decide on each trial whether their response felt rather easy or rather difficult, without any reference to which cue they had to use. Both prime and blank duration lasted for 23 ms. The main difference with Experiment 1 was that the task was alternatingly performed in either a context with 80% congruent trials and in a context with 20% congruent trials. Variance caused by this manipulation was modelled as a random variable, but not included as fixed factor. Results showed that congruent trials were more frequently judged to be easy (*M* = 85.7%) than incongruent trials (*M* = 80.01%), $\chi2(1) = 4.01$, $p = .045$. Also, the

proportion of 'easy' responses was higher on response repetitions (*M* = 84.9%), compared to response alternations (*M* = 81.4%), $\chi 2(1) = 4.38$, $p = .036$. Finally, the proportion of 'easy' judgments increased with increasing RTs, $\chi 2(1) = 82.05$, $p < .001$. There were no significant interactions, all *p's* > .15.

Finally, we reanalyzed a fourth unpublished dataset (*N* = 70) from a study similar to Experiment 1, except that participants indicated their judgment of difficulty on a four point scale. To be consistent with the analysis of Experiment 1, judgments 1 and 2 (i.e., 'conflicting' and 'guess conflicting', respectively) were taken together and judgments 3 and 4 (i.e., 'guess non-conflicting' and 'non conflicting', respectively) were taken together. Moreover, in addition to congruent and incongruent trials, the experiment contained neutral trials in which the prime arrow pointed both rightwards and leftwards. Importantly, the instructions in this study remained neutral with respect to which cue participants had to focus on. The data of one participant was excluded because mean accuracy was below 80%, and that of two other participants because their median RT of correct trials was more than 3 SDs above the group mean. The results showed a main effect of congruency, $\chi 2(2) = 122.03$, $p < .001$. Congruent trials were more frequently judged to be easy (*M* = 95.7%) than incongruent trials (*M* = 93.7%), $\chi 2(1) = 89.53$, $p < .001$, but congruent trials did not differ from neutral trials (*M* = 95.7%), $\chi 2(1) = 0.40$, $p = .52$. Also, the proportion of 'easy' responses was higher on response repetitions (*M* = 95.9%), compared to response alternations (*M* = 94.1%), $\chi 2(1) = 17.56$, $p < .001$. Finally, the proportion of 'easy' judgments increased with increasing RTs, $\chi 2(1) = 108.48$, $p < .001$. Interestingly, there also was a significant interaction between response repetition and reaction time, $\chi 2(1) = 5.42$, $p = .020$, showing that the effect of reaction time on the subjective difficulty judgment was slightly larger on response alternations compared to response repetitions. Finally, there was a significant three-way interaction, $\chi 2(2) = 6.87$, $p = .032$, reflecting that this interaction between response repetition and reaction time was only significant for congruent trials, $\chi 2(1) = 8.80$, $p = .003$, but not for neutral or incongruent trials, both *p*'s > .42. No other effects were significant, all *p*'s > .25.

References

1. Desender, K., Van Opstal, F. & Van den Bussche, E. Feeling the Conflict: The Crucial Role of Conflict Experience in Adaptation. *Psychol. Sci.* **6,** 375–83 (2014).

2. Instructions of Experiments 2a and 2b

Below, translated versions (from Dutch) of the exact instructions of Experiments 2a and 2b can be found. Note that these instructions were provided after participants performed the priming task and the detection task (see Figure 3), so they were already familiar with the priming task and were informed that invisible prime arrows were presented during the detection task.

Experiment 2a

As you have probably figured out, before the large arrow that you had to respond to, we always presented another small arrow. This arrow was presented so briefly that you probably never noticed its presence, but it nevertheless might have affected your performance. This small arrow can either point in the same direction as the large arrow or in the other direction. Because of this, your response to the large arrow might have felt rather easy (when both arrows pointed in the same direction) or rather difficult (when both pointed in a different direction). Importantly, it is possible to consciously notice this experience of difficulty, even though you never consciously saw the small arrow. In this part of the experiment, we want to train you to detect these subtle differences in difficulty based on the conflict between the two arrows. Put differently, we are going to train you to become an expert in detecting differences in difficulty caused by the compatibility between the two arrows.

During the experiment, the small (invisible) arrow will either point in the same or in a different direction compared to the large arrow. Normally, it should be possible for you to detect whether both are pointing in the same or in a different direction on a trial, based on your experience as to whether it felt rather easy or rather difficult to respond to the large arrow on that trial. When both arrows point in the same direction, you might experience that it felt easy to respond, and that you only wanted to press with one hand. When both arrows point in a different direction, you might experience that you also had the urge to press with the incorrect hand, even though you pressed correctly. Beware, this difference can be very small, so try to be very sensitive to the least noticeably differences in difficulty.

From now on you always have to perform two tasks: 1. Respond to the direction of the large arrow: left = D, right = K. 2. Indicate how much difficulty you experienced when responding to the large arrow. Rather much difficulty: press up. Rather less difficulty: press down. Importantly, try to use both response options equally frequent.

Experiment 2b

As you might have noticed, sometimes it feels quite difficult and sometimes quite easy to respond fast and correctly to the direction of the arrow. One of the reasons for this feeling is the speed with which you are able to respond. On some trials you will be able to respond fast to the direction of the arrow, due to which these trials might feel easy. On other trials you will respond rather slowly, due to which your response on those trials might feel more difficult. In this part of the experiment, we want to train you to detect these subtle differences in difficulty based on your

reaction time. Put differently, we are going to train you to become an expert in detecting differences in difficulty caused by differences in your reaction time.

During the experiment, on each trial you will have to indicate how much difficulty you experienced to respond fast and correctly on that trial. When providing this judgment, please try to mainly base this judgment on the speed with which you responded. Beware, this difference can be very small, so try to be very sensitive to the least noticeably differences in reaction time. You might be able to feel it was rather easy to respond because you responded faster than average. Alternatively, you might be able to feel it was rather difficult to respond because you responded slower than average. Because speed is relative, try to use your own mean response time as a reference point.

From now on you always have to perform two tasks: 1. Respond to the direction of the large arrow: left = D, right = K. 2. Indicate how much difficulty you experienced when responding to the large arrow. Rather much difficulty: press up. Rather less difficulty: press down. Importantly, try to use both response options equally frequent.
